# Pre-COVID-19 short sleep duration and eveningness chronotype are associated with incident suicidal ideation during COVID-19 pandemic in medical students: a retrospective cohort study

**DOI:** 10.3389/fpubh.2024.1406396

**Published:** 2024-07-23

**Authors:** Dandan Zheng, Qingsong Qin, Yingyin Peng, Hao Zhong, Yerui Huang, Hongjie Wang, Qiqing Tan, Yun Li

**Affiliations:** ^1^Department of Sleep Medicine, Mental Health Center of Shantou University, Shantou, Guangdong, China; ^2^Sleep Medicine Center, Shantou University Medical College, Shantou, Guangdong, China; ^3^Faculty of Medicine of University of Manitoba Joint Laboratory of Biological Psychiatry, Shantou University Medical College, Shantou, Guangdong, China; ^4^Laboratory of Human Virology and Oncology, Shantou University Medical College, Shantou, Guangdong, China

**Keywords:** short sleep duration, chronotype, suicidal ideation, COVID-19, medical students

## Abstract

**Introduction:**

Cross-sectional evidence suggests that sleep problems increased the risk of suicide during the 2019 coronavirus disease (COVID-19) pandemic. However, a lack of longitudinal studies examined the relationship between pre-COVID-19 sleep duration, chronotype and incident suicide during the COVID-19 pandemic. Thus, we examined these associations in a longitudinal study of medical students.

**Methods:**

From the Shantou College Student Sleep Cohort, a total of 333 first and second grade medical students (age 19.41 ± 0.82 years, female 61.26%), without suicidal ideation (SI) at pre-COVID-19 period, were followed up during the COVID-19 pandemic. Incident SI was defined by their response to the 9^th^ question from the Beck Depression Inventory. Short sleep duration was defined as less than 7 h/night. The Morningness-Eveningness Questionnaire was used to evaluate the participants’ chronotype. Logistic regression with adjusted odds ratios (AOR) and 95% confidence intervals (95% CI) was used to examine the association between sleep and SI.

**Results:**

The incidence of SI during the COVID-19 pandemic was 5.71%. Logistic regressions with confounding factors adjustment showed that both short sleep duration (AOR = 4.91, 95% CI = 1.16–20.74) and eveningness (AOR = 3.80, 95% CI = 1.08–13.30) in the pre-COVID-19 period were associated with increased risk of incident SI during the COVID-19 pandemic.

**Conclusion:**

Pre-COVID-19 short sleep duration and eveningness predict incident SI during the COVID-19 pandemic in medical students. Prolonging sleep duration may help to decrease SI during major public health crises.

## 1 Introduction

Suicide is one of the leading causes of death worldwide. Based on the data from the World Health Organization, suicide is the fourth leading cause of death in adolescents and young people (15–29 years) (1). Suicidal ideation (SI) is a strong predictor of suicide attempts and completed suicide (2). In college students, the 1-year incidence of SI ranges from 3.00 to 8.53% worldwide (3, 4) and the prevalence of SI has increased by 138% in 10 years (2007–2017) (5). It is important to identify risk factors of SI within this vulnerable age group, which is critical to its prevention and early intervention.

### 1.1 SI and short sleep duration

According to the National Sleep Foundation’s sleep duration recommendations, young adults (18–25 years old) sleep 7 to 9 h per day is recommended (6). However, college students, especially in South Asian countries and China, always have heavy course schedules and poor time management (7). A previous study showed that nearly 46% of Chinese college students slept less than 7 h (8). Short sleep duration is associated with worse health conditions (9) and higher all-cause mortality risk (10). Growing evidence suggests that short sleep duration is a potentially salient risk factor for SI in adolescents and young adults (11–14). Longitudinal studies consistently found that short sleep duration is associated with a 1.5- to 3-fold increased incidence of SI in adolescents and young adults (15, 16).

### 1.2 SI and chronotype

Chronotype is usually described as morningness, intermediateness, and eveningness. Each chronotype is associated with different personality traits. For example, eveningness shows more impulsiveness than morningness (17). The risk of suicidality, including suicidal thoughts and attempts, is higher in the eveningness chronotype than the other chronotypes among adolescents (18). A cross-sectional study showed that eveningness subjects independently increased 3.87-fold SI in Chinese young adults (19). However, a 3-year follow-up longitudinal study showed that a persistent eveningness chronotype was not associated with an increased risk of SI in adolescents (20). Taken together, the association between the chronotype and SI is not clear yet.

### 1.3 SI during the 2019 coronavirus disease pandemic

The 2019 coronavirus disease (COVID-19) started in December 2019 and became a global pandemic 1 month later. COVID-19 continued to cause death and disrupt millions of lives and commerce, and increased sleep and psychological problems, are all known risk factors for suicide (21). Specifically, several studies reported that the prevalence of SI during COVID-19 was higher than in the pre-pandemic period in college students (22–24). Because of the public health policy, college students had to change their conventional learning mode, such as stopping on-campus learning and clinical rotations and taking online classes instead. Due to these changes, many medical students had barriers finding their worth in health care and worried about their graduations (25). Medical students were a group of vulnerable population with a higher likelihood of SI during the COVID-19 pandemic (26).

A few studies suggested that short sleep and eveningness are associated with an increased risk of SI (15, 19). However, most evidence is based on cross-sectional studies, whereas no longitudinal study has examined the relationship between pre-COVID-19 sleep and suicide risk during the COVID-19 pandemic. To fill this gap, we examined these longitudinal associations. We hypothesized that habitual pre-COVID-19 short sleep duration and eveningness chronotype are associated with the increased risk of incident SI during the COVID-19 pandemic in medical students.

## 2 Methods

### 2.1 Study population

**Figure 1:**
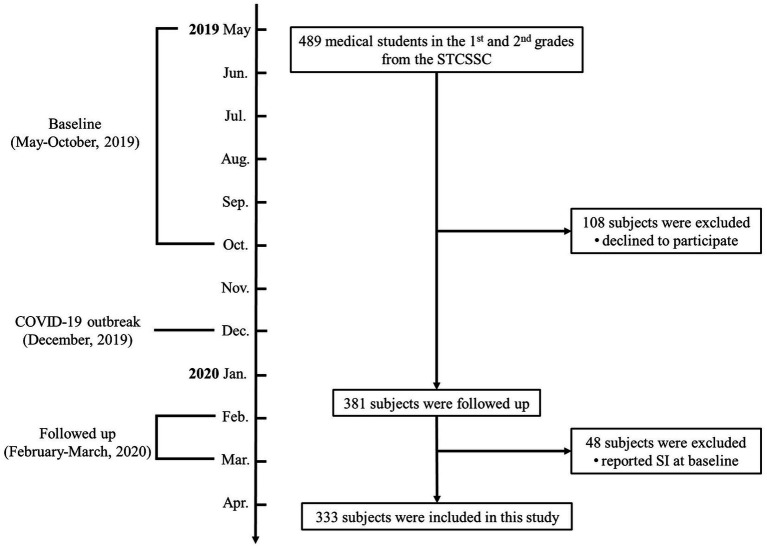
The flowchart of this study. SI, suicidal ideation; STCSSC, Shantou College Student Sleep Cohort.

The data presented here were collected from the Shantou College Student Sleep Cohort (ChiCTR2100051755) (27). To determine baseline, 489 Shantou University medical students in the first and the second grades volunteered to complete a series of online questionnaires related to sleep and mood, through an online Questionnaire Star program, in early May and late October of 2019, during the pre-COVID-19 pandemic. Medical students in the Shantou University begin their psychiatry courses and clinical practical rotations in hospitals in their 3^rd^ academic year. Because the professional medical knowledge of psychiatry may affect the response to the questionnaires for assessing depressive and anxiety symptoms, as well as hospital rotations may affect their sleep pattern, we did not include the junior, senior and fifth-year medical students in this study. Among the included 489 freshmen and sophomores at baseline, 381 were followed up (follow-up rate of 78%) during the COVID-19 pandemic in China between February to March of 2020. During this period, all included students were staying at home with extended winter holidays due to the COVID-19 pandemic. After excluding 48 subjects with SI at baseline, a total of 333 subjects were included in this study. This study was approved by the Research Ethics Board of Shantou University Mental Health Center (SUMHC20190510). All participants signed informed consent online and were informed that their personal information would be confidential. Figure 1 depicts the flow of this study.

Because no longitudinal study examined the association between baseline eveningness chronotype and SI, the sample size calculation of the present student population was based on the incidence of SI (range from 7.60 to 17.26%) among general adolescents (15, 16), and the odds ratio (OR) for SI according to short sleep duration (OR = 3.78). At the power of 0.9, and a significance level of 0.05, the sample size needed was calculated to be between 114 to 204 subjects. In our study, we included 381 subjects, which had enough power to test our hypothesis. The sample size determination was conducted using the PASS 15.0.5 program.

### 2.2 Measures

#### 2.2.1 Sleep assessments

The Pittsburgh Sleep Quality Index (PSQI) is a self-assessment questionnaire for assessing the subject’s habitual sleep quality (28). It consists of 19 items to assess 7 components of sleep, including subjective sleep quality, sleep latency, sleep duration, sleep efficiency, sleep disturbance, sleep medication condition, and daytime dysfunction. The total score of PSQI ranges from 0 to 21, higher PSQI scores indicate worse sleep quality (28). The Chinese version of the PSQI has good validity and reliability (Cronbach’s alpha = 0.78) (29). The total sleep time (TST) was extracted from the PSQI based on the question “During the past month, how many hours of actual sleep did you get each night.” According to the recommendation of the National Sleep Foundation for young adults aged 18–25 years old, short sleep duration was defined as a total sleep duration less than 7 h in our study (6). In addition, bedtime, wake up time, time in bed (TIB), and sleep onset latency (SOL) were also extracted from the PSQI. Sleep efficiency was calculated based on (TST/TIB) * 100 (%).

The Morningness-Eveningness Questionnaire (MEQ) consists of 19 items to evaluate subject’s circadian rhythms incorporating sleep and awake preference (30). The total score of MEQ ranges from 16 to 86, higher total scores of MEQ indicate stronger morningness preference. The Chinese version of the MEQ has good validity and reliability (Cronbach’s alpha = 0.77) (31). In this study, eveningness chronotype was defined as a score between 16 and 41, intermediateness chronotype was defined as an MEQ score between 42 and 58, and morningness chronotype was defined as an MEQ score between 59 and 86.

#### 2.2.2 Depressive symptoms and SI

The Beck Depression Inventory (BDI) consists of 21 items to assess subject’s depression symptoms (32). The total score of BDI ranges from 0 to 63, higher total scores of BDI indicate more severe depressive symptoms. The Chinese version of the BDI has good validity and reliability (Cronbach’s alpha = 0.86) (33). SI was evaluated based on the subject’s response to the 9^th^ item of the BDI. If a subject responds “I do not have any thoughts of killing myself,” he or she would be classified as without SI; while if a subject responds “I have thoughts of killing myself, but I would not carry them out” or “I would like to kill myself” or “I would kill myself if I had the chance,” he or she would be classified as SI in this study.

#### 2.2.3 Anxiety symptoms

The Beck Anxiety Inventory (BAI) consists of 21 items to assess subject’s anxiety symptoms (34). The total score of BAI ranges from 0 to 63, higher total scores of BAI indicate more anxiety symptoms. The Chinese version of the BAI has good validity and reliability (Cronbach’s Alpha = 0.94) (35).

#### 2.2.4 Other measurements

Sociodemographic characteristics, such as age, sex, self-reported height and weight, satisfaction with the financial situation, pre-bedtime prolonged electronic screen media use for entertainment (PESM-E), caffeine consumption and medication use were also collected. Body mass index (BMI) was calculated based on weight/height^2^ (kg/m^2^). Financial status was collected from a self-report question, “Are you satisfied with your financial situation?”. Subjects who answered “yes” would be classified as satisfied with their financial situation. Pre-bedtime PRSM-E was defined as using electronic screen media for entertainment longer than 60 min/night after 10:00 p.m. during the past 6 months. Caffeine consumption was defined as drinking coffee, tea, cola and/or caffeinated energy drinks on most days. Medication use was defined as taking any medication during the past 3 months.

### 2.3 Statistical analysis

Data are presented as mean ± standard deviation (SD) for continuous variables, and percentage for categorical variables. Kolmogorov–Smirnov test was used for normality testing. Comparisons between groups were performed using an independent *t*-test, Mann–Whitney *U*-test, or Kruskal–Wallis test for normally and non-normally distributed continuous variables, respectively, or using the *χ*^2^ test for categorical variables. To examine the association of pre-COVID-19 pandemic short sleep duration and eveningness chronotype with incident SI during the COVID-19 pandemic in China, logical regression with pre-COVID-19 short sleep duration and eveningness chronotype as predictors and incident SI during the COVID-19 pandemic in China as an outcome were conducted. In the regression models, we adjusted for age, sex, BMI, and variables with between-group differences in Table 1. Considering that levels of depressive and anxiety symptoms at follow-up may also affect the risk of incident SI, we also adjusted the changes of the total scores of BAI and BDI (follow-up score minus baseline score) instead of the corresponding baseline scores in secondary analysis. All hypothesis tests were 2-sided. Adjusted odds ratios (AOR) and 95% confidence intervals (95% CI) were presented. A *p*-value<0.05 was used to determine statistical significance. All statistical analyses were performed using IBM SPSS 26.0.

**Table 1:** Demographic, sleep and mood characteristics of the included subjects at pre-COVID-19 period.

Variables	Overall (*n* = 333)	Incident suicidal ideation		*P*-value
		No (*n* = 314)	Yes (*n* = 19)	
Demographic characteristic				
Age, years	19.41 ± 0.82	19.39 ± 0.82	19.59 ± 0.82	0.379
BMI, kg/m^2^	20.27 ± 2.52	20.19 ± 2.46	21.61 ± 3.08	**0.031**
Sex (Female)	204 (61.26)	193 (61.46)	11 (57.89)	0.756
Grade (Freshman)	262 (78.68)	249 (79.30)	13 (68.42)	0.403
Satisfied with the financial situation	274 (82.28)	260 (82.80)	14 (73.68)	0.483
Pre-bedtime PESM-E	179 (53.75)	171 (54.46)	8 (42.11)	0.294
Caffeine consumption	175 (52.55)	166 (52.87)	9 (47.37)	0.641
Medication use	84 (25.23)	80 (25.48)	4 (21.05)	0.873
Sleep characteristics				
PSQI, points	4.43 ± 2.18	4.36 ± 2.12	5.58 ± 2.85	0.099
Short sleep duration	167 (50.15)	151 (48.09)	16 (84.21)	**0.002**
Bedtime	23:52 ± 0:34	23:51 ± 0:34	24:07 ± 0:33	**0.039**
Wake up time	07:06 ± 0:23	07:06 ± 0:23	07:12 ± 0:18	0.268
TIB, hours	7.24 ± 0.66	7.25 ± 0.66	7.07 ± 0.60	0.231
SOL > 30 min	29 (8.71)	25 (7.96)	4 (21.05)	0.122
Sleep efficiency, %	91.06 ± 7.77	91.15 ± 7.74	89.61 ± 8.28	0.388
Chronotype				
Morningness	39 (11.71)	37 (11.78)	2 (10.53)	**0.043**
Intermediateness	263 (78.98)	251 (79.94)	12 (63.16)	
Eveningness	31 (9.31)	26 (8.28)	5 (26.32)	
Mood characteristics				
BAI, points	5.05 ± 5.49	4.85 ± 5.49	8.32 ± 4.52	**<0.001**
BDI, points	7.61 ± 6.20	7.33 ± 6.09	12.21 ± 6.29	**0.001**

BAI, Beck Anxiety Inventory; BDI, Beck Depression Inventory; BMI, body mass index; PESM-E, prolonged electronic screen media use for entertainment; PSQI, Pittsburgh Sleep Quality Index; SOL, sleep onset latency; TIB, time in bed. *p*-values in bold (*p* < 0.05) indicated a statistically significant difference.

## 3 Results

A total of 381 subjects were followed up during the COVID-19 pandemic, a follow-up rate of 78%, and the median follow-up duration was 9 months. There were no significant differences in baseline age, sex and BMI between the total subjects and the subjects who were followed up (all *p*-value ≥0.05). Among the 333 subjects who did not have SI in the pre-COVID-19 pandemic period, 19 subjects (5.71%) reported incident SI during the COVID-19 pandemic. Table 1 presents the demographic, sleep, and mood characteristics of the entire sample pre-COVID-19 and stratified by the presence or absence of incident SI during the COVID-19 pandemic.

As shown in Table 1, pre-COVID-19 BMI [21.61 ± 3.08 kg/m^2^ versus (vs.) 20.19 ± 2.46 kg/m^2^, *p* = 0.031], bedtime (24:07±0:33 vs. 23:51±0:34, *p* = 0.039), the proportion of self-reported short sleep duration (84.21% vs. 48.09%, *p* = 0.002), eveningness chronotype (26.32% vs. 8.28%, *p* = 0.043), and the total scores of BAI (8.32 ± 4.52 points vs. 4.85 ± 5.49 points, *p* < 0.001) as well as BDI (12.21 ± 6.29 points vs. 7.33 ± 6.09 points, *p* = 0.001) were significantly higher in students with incident SI compared to those without incident SI during the COVID-19 period.

**Figure 2:**
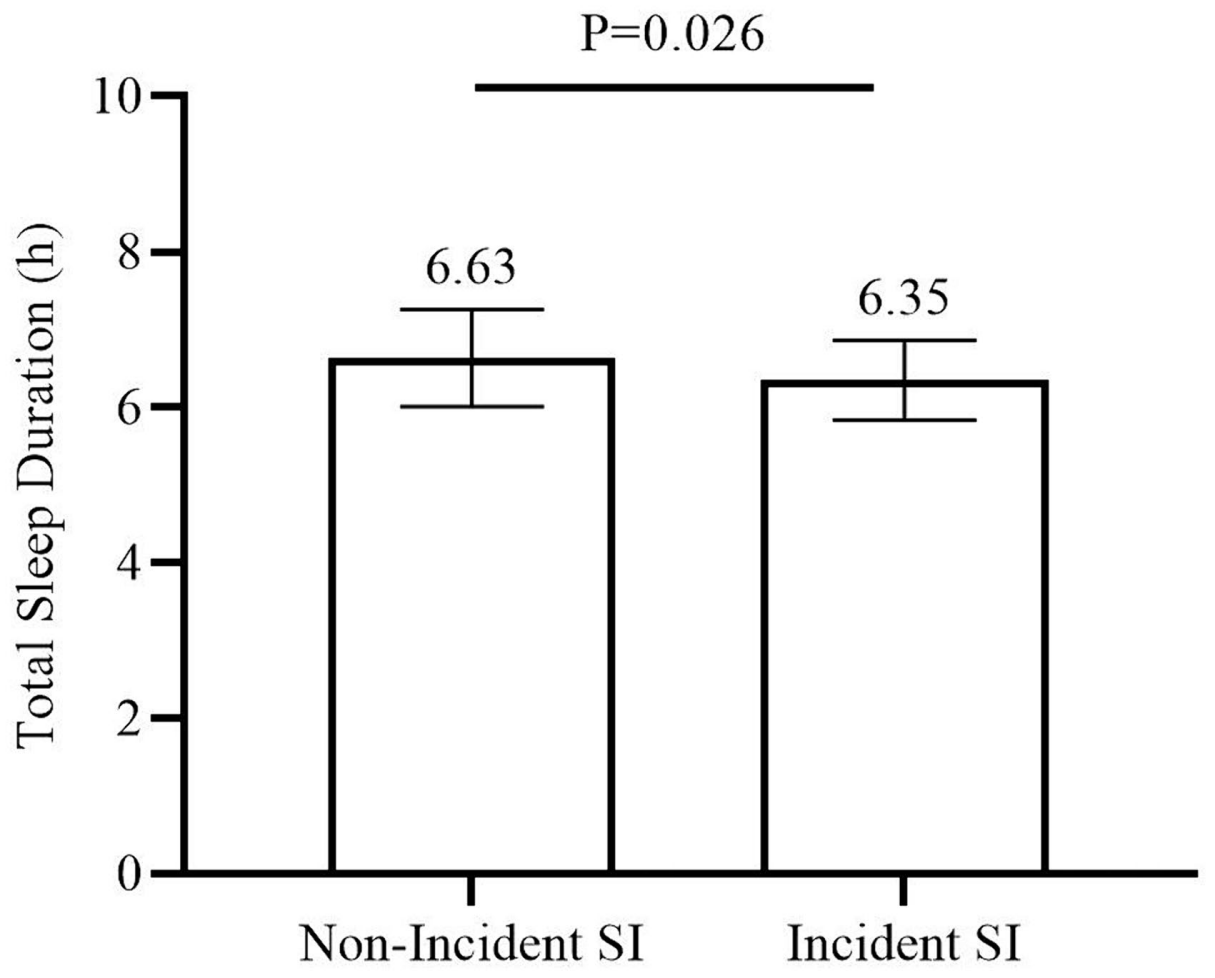
Comparison in total sleep duration between subjects with or without incident SI. Comparison in total sleep duration between subjects with or without incident SI during the COVID-19 pandemic by using independent *t*-test. Means with error bars (SD) of the sleep duration are shown for each group. SD, standard error; SI, suicidal ideation.

**Figure 3:**
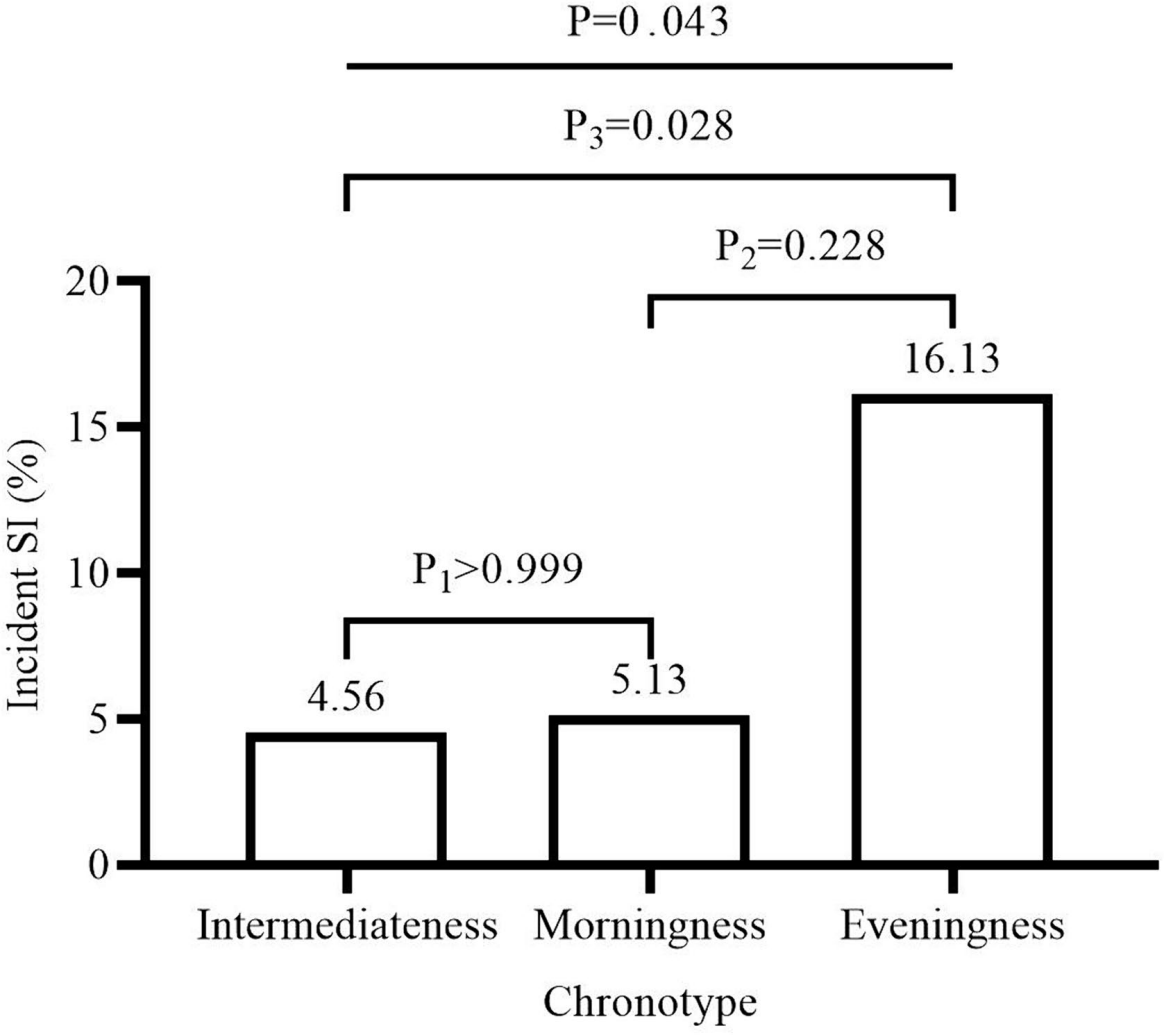
Proportion of incident SI in different chronotypes. Comparison of the proportions of incident SI in each chronotype group by using *χ*^2^ test. P_1_: Morningness vs. Intermediateness. P_2_: Morningness vs. Eveningness. P_3_: Intermediateness vs. Eveningness. SI, suicidal ideation.

We found that pre-COVID-19 short sleep duration (AOR = 4.91, 95% CI = 1.16–20.74, *p* = 0.031) and pre-COVID-19 eveningness chronotype (AOR = 3.80, 95% CI = 1.08–13.30, *p* = 0.037) were associated with increased risk of incident SI during the COVID-19 pandemic, after adjusting for baseline age, bedtime, BMI, sex, and total scores of BAI and BDI (Model 1 in Table 2). In secondary analysis, when we used the changes of BAI and BDI from follow-up to baseline, instead of the total scores of baseline BAI and BDI as covariates in Model 2 (Table 2), the results were similar and significant (short sleep duration: AOR = 6.79, 95% CI = 1.41–32.74, *p* = 0.017; eveningness chronotype: AOR = 5.59, 95% CI = 1.43–21.79, *p* = 0.013). However, the morningness chronotype was not associated with incident SI (AOR = 3.28, 95% CI = 0.57–18.79, *p* = 0.183). As shown in Figure 2, students who had incident SI during the COVID-19 pandemic had shorter sleep duration compared to those who did not have incident SI (6.35 h vs. 6.63 h, *p* = 0.026). Figure 3 depicts the comparisons of the proportion of SI among the three chronotypes (Intermediateness 4.56% vs. Morningness 5.13% vs. Eveningness 16.13%, *p* = 0.043). Moreover, we examined the association between the baseline score of PSQI, instead of short sleep duration, and incident SI. PSQI was not associated with an increased risk of incident SI during the COVID-19 pandemic (AOR = 1.07, 95% CI = 0.84–1.36, *p* = 0.585).

**Table 2:** The association between baseline short sleep duration, eveningness chronotype and incident SI.

Variable	Model 1		Model 2	
	AOR (95%CI)	*P*-value	AOR (95%CI)	*P*-value
Total sleep time ≥ 7 h	Ref		Ref	
Total sleep time < 7 h	4.91 (1.16–20.74)	**0.031**	6.79 (1.41–32.74)	**0.017**
Intermediateness chronotype	ref		ref	
Morningness chronotype	3.28 (0.57–18.79)	0.183	1.18 (0.14–9.73)	0.881
Eveningness chronotype	3.80 (1.08–13.30)	**0.037**	5.59 (1.43–21.79)	**0.013**

Model 1 was adjusted for baseline age, bedtime, BMI, sex, and total scores of BAI and BDI. Model 2 was adjusted for baseline age, bedtime, BMI, changes of BAI and BDI from follow-up to baseline, and sex. *P*-values in bold (*P* < 0.05) indicated a statistically significant difference.

Supplementary Tables S1, S2 present the sociodemographic, sleep and mood characteristics of students stratified by sleep duration and chronotypes, respectively. Students with short sleep duration had higher proportion of pre-bedtime PESM-E (59.88% vs. 47.59%, *p* = 0.025), later bedtime (24:08 ± 0:32 vs. 23:36 ± 0:28, *p* < 0.001), and shorter TIB (6.98 ± 0.66 h vs. 7.49 ± 0.56 h, *p* < 0.001), whereas no significant difference was found in wake up time (07:07 ± 0:22 vs. 07:05 ± 0:23, *p* = 0.900) or sleep onset latency (8.98% vs. 8.43%, *p* = 0.859) (Supplementary Table S1). Furthermore, the proportion of pre-bedtime PESM-E (Intermediateness 53.23% vs. Morningness 38.46% vs. Eveningness 77.42%, *p* = 0.005), bedtime (Intermediateness 23:53 ± 0:34 vs. Morningness 23:29 ± 0:26 vs. Eveningness 24:08 ± 0:28, *p* < 0.001), and TIB (Intermediateness 7.23 ± 0.66 h vs. Morningness 7.52 ± 0.62 h vs. Eveningness 6.99 ± 0.59 h, *p* = 0.001) were significantly different among three chronotypes. Wake up time (Intermediateness 07:07 ± 0:23 vs. Morningness 07:00 ± 0:21 vs. Eveningness 07:08 ± 0:22, *p* = 0.100), the proportion of short sleep duration (Intermediateness 52.09% vs. Morningness 33.33% vs. Eveningness 54.84%, *p* = 0.079) and SOL (Intermediateness 8.75% vs. Morningness 2.56% vs. Eveningness 16.13%, *p* = 0.151) showed no statistical difference among different chronotypes (Supplementary Table S2).

## 4 Discussion

In adolescents and young adults, suicide is one of the leading causes of death (1). Sleep disturbance has been known as a potential risk factor for suicide (36). The prevalence of SI and sleep disturbance both increased during the COVID-19 pandemic (22–24, 37, 38). This is the first study to examine the association between the pre-COVID-19 short sleep duration, eveningness chronotype and incident SI during the COVID-19 pandemic in medical students. Our findings suggest that pre-COVID-19 short sleep duration and eveningness chronotype are significantly associated with an increased risk of incident SI during the COVID-19 pandemic in medical students.

Psychological problems associated with COVID-19, including suicide, have drawn much attention from the public, as well as the medical community (39). Several studies have suggested that incident SI is associated with short sleep duration during the COVID-19 pandemic in adolescents (15, 40). However, no longitudinal study has examined the association between pre-COVID-19 sleep duration and incident SI during the COVID-19 period. In the current study, our findings suggested that medical students who slept less than 7 h per night had a higher risk of incident SI during the COVID-19 pandemic. In our study, the students with short sleep duration had later bedtime and shorter TIB, but not more difficulty in getting or maintaining. Furthermore, we found that the students with short sleep duration spent longer time on electronic screen media entertainment before bedtime. It needs to be noted that each medical student needs to attend the fixed early morning class at 8:00 a.m. in our study. Taken together, it appears that short sleep duration in our sample is mainly due to lake of sleep opportunities, which is consistent with a previous study conducted in China (41). In our study, electronic screen media entertainment was one of the leading reasons for delayed bedtime for medical students. All this evidence suggests that prolonging sleep by increasing sleep opportunities (e.g., advance bedtime or delay school time) might be helpful (42, 43). Moreover, our finding of short sleep duration associated with incident SI is consistent with previous longitudinal studies that were not conducted during the COVID-19 pandemic (11, 13). Taken together, it appears that self-reported short sleep duration is a predictor for future SI, no matter whether in common situations or under major health events.

The mechanisms for short sleep duration with increased risk of SI remain unclear. First, it might be associated with inflammation. Previous findings have suggested that insufficient sleep is associated with inflammation (44−46). Inflammation increases the risk of depression, which is one of the strong predictors of SI (47). Second, it might be associated with decreased serotonergic activity. It has been reported that short sleep is associated with decreased serotonergic activity. Lower concentrations of serotonin have been reported to be associated with suicide (48). However, we do not have biological data enabling us to examine the underlying mechanisms for the association of short sleep duration with a higher risk of SI. Future studies should examine the mechanisms linking the association between short sleep duration and SI.

An individual’s preference for the time of sleeping and being awake is called their chronotype. Those with an eveningness chronotype are likely to go to bed at midnight, wake later, and show high efficiency and activity in the afternoon compared to those with the morningness chronotype. In the current study, we found that students with the eveningness chronotype have a higher risk of incident SI even after adjusting for depression and short sleep duration. In our study, we found eveningness chronotype, independent of short sleep duration, predicted the incidence of SI during COVID-19. This finding is consistent with some studies that were conducted in patients with depressive disorder (49, 50). However, our finding is inconsistent with a previous longitudinal study that was conducted in general adolescents (20). The inconsistent findings might be due to that study investigated individuals with a persistent eveningness chronotype, while our study used baseline eveningness chronotype as a predictor of incident SI. Furthermore, it has been reported that both suicidality and eveningness have genetic variations in the ARNTL and CLOCK genes (51). It appears that suicidality may share the same genetic variations with eveningness.

It needs to be noted that the findings of this study were based on self-reported sleep duration. Self-reported sleep duration estimates are about 0.5–1 h longer than objective measures in general population (52–54). Self-reported sleep duration does not only reflect sleep duration *per se*, but is also associated with other factors, such as age, sex, socioeconomic status, education, race, sleep quality, physical health problems (e.g., pain and obesity) and mental health problems (e.g., depression and emotional stress) (55). In the current study, when we further controlled the potential confounders that may affect self-reported sleep duration, the results remained similar and significant.

## 5 Clinical implications

Our findings suggest that pre-COVID-19 short sleep duration and the eveningness chronotype are risk factors for incident SI during the COVID-19 pandemic. In our study, we found self-reported short sleep duration was caused by the later bedtime rather than insomnia symptoms. Sleep is a modifiable factor that can reduce suicide risk and be targeted through public health prevention. Prolonged sleep has been shown to be associated with a decreased risk of SI (56). In Colorado, students’ sleep duration increased by 4.6 min for the school start time delaying 15 min, and the risk of suicidal attempts was also decreased (57). It appears that earlier bedtime for prolonging sleep duration or more flexible time schedules for eveningness may help to decrease incident SI in medical students. More multi-center longitudinal, intervention and experimental studies are needed to further confirm the association between short sleep duration, eveningness chronotype and SI, as well as their underlying mechanisms.

## 6 Strengths and limitations

Strengths of this study include the prospective design and careful control for multiple potential confounders that may affect the association between sleep duration, chronotype and SI. Several limitations should be acknowledged and taken into account when interpreting our results. First, given that different majors may affect the understanding of symptoms of depression and anxiety, and be affected differently by the COVID-19 pandemic (58), we only included medical students. Thus, our results cannot be generalized to other populations. The association of sleep duration, chronotype and risk of SI in the general population needs to be examined in future studies. Second, our findings were based on self-report questionnaires, which may lead to recall bias. However, it has been reported that the questionnaires used in this study are valid and reliable for the Chinese population.

## 7 Conclusion

Our findings suggest that pre-COVID-19 self-reported short sleep duration and eveningness chronotype predict incident SI during the COVID-19 pandemic in medical students. Prolonging sleep duration by earlier bedtime or flexible school time may help to decrease SI in medical students during major public health crises. However, self-reported sleep might not fully reflect objective sleep states and we only included medical students in one college, restricting our findings to generalize to other populations. Future multi-center studies using objective sleep measurements are needed in the general population.

## Data availability statement

The datasets used and/or analyzed in the current study are available from the corresponding author on reasonable request.

## Ethics statement

The studies involving humans were approved by Research Ethics Board of Shantou University Mental Health Center. The studies were conducted in accordance with the local legislation and institutional requirements. The participants provided their written informed consent to participate in this study.

## Author contributions

DZ: Conceptualization, Data curation, Formal analysis, Methodology, Writing – original draft, Writing – review & editing. QQ: Conceptualization, Supervision, Writing – review & editing. YP: Conceptualization, Data curation, Investigation, Writing – review & editing. HZ: Conceptualization, Data curation, Investigation, Writing – review & editing. YH: Conceptualization, Data curation, Investigation, Writing – review & editing. HW: Conceptualization, Data curation, Investigation, Writing – review & editing. QT: Conceptualization, Data curation, Investigation, Writing – review & editing. YL: Conceptualization, Funding acquisition, Supervision, Writing – review & editing.
