# Supplementary material for: Pre-COVID-19 short sleep duration and eveningness chronotype are associated with incident suicidal ideation during COVID-19 pandemic in medical students: a retrospective cohort study
Source: Front Public Health. 2024 Jul 23;12:1406396. doi: 10.3389/fpubh.2024.1406396 (PMC11300336; doi:10.3389/fpubh.2024.1406396)
Supplement: Supplementary file 1 [file Table_1.DOCX]

Supplementary Material

# Supplementary Tables

Supplementary Table 1. Baseline sample characteristics stratified by sleep duration.

| Variables | Sleep Duration | | P value |
| --- | --- | --- | --- |
|  | ≥7h (n=166) | <7h (n=167) |  |
| **Demographic characteristic** | | | |
| Age, years | 19.30±0.84 | 19.51±0.78 | **0.021** |
| BMI, kg/m^2^ | 20.22±2.60 | 20.31±2.44 | 0.728 |
| Sex (Female) | 89 (53.61) | 115 (68.86) | **0.004** |
| Grade (Freshman) | 139 (83.73) | 123 (73.65) | **0.025** |
| Satisfied with the financial situation | 142 (85.54) | 132 (79.04) | 0.120 |
| Caffeine consumption | 85 (51.20) | 90 (53.89) | 0.623 |
| Medication use | 36 (21.69) | 48 (28.74) | 0.138 |
| Pre-bedtime PESM-E | 79 (47.59) | 100 (59.88) | **0.025** |
| **Sleep characteristics** | | | |
| PSQI, points | 3.55±1.62 | 5.31±2.30 | **<0.001** |
| Bedtime | 23:36±0:28 | 24:08±0:32 | **<0.001** |
| Wake up time | 07:05±0:23 | 07:07±0:22 | 0.900 |
| TIB, hours | 7.49±0.56 | 6.98±0.66 | **<0.001** |
| Sleep efficiency, % | 94.25±5.66 | 87.90±8.28 | **<0.001** |
| SOL>30 min | 14 (8.43) | 15 (8.98) | 0.859 |
| Chronotype | | | |
| Morningness | 26 (15.66) | 13 (7.78) | 0.079 |
| Intermediateness | 126 (75.90) | 137 (82.04) |  |
| Eveningness | 14 (8.43) | 17 (10.18) |  |
| **Mood characteristics** | | | |
| BAI, points | 3.81±4.07 | 6.27±6.39 | **<0.001** |
| BDI, points | 6.47±6.11 | 8.74±6.10 | **<0.001** |

Abbreviations: BAI, Beck Anxiety Inventory; BDI, Beck Depression Inventory; BMI, body mass index; PESM-E, prolonged electronic screen media use for entertainment; PSQI, Pittsburgh Sleep Quality Index; SOL, sleep onset latency; TIB, time in bed. P-values in bold (P<0.05) indicated a statistically significant difference.

Supplementary Table 2. Baseline sample characteristics stratified by chronotype.

| Variables | Chronotype | | | Overall P | P_1_ | P_2_ | P_3_ |
| --- | --- | --- | --- | --- | --- | --- | --- |
|  | Intermediateness (n=263) | Morningness (n=39) | Eveningness (n=31) |  |  |  |  |
| **Demographic characteristic** | | | | | | | |
| Age, years | 19.38±0.82 | 19.23±0.69 | 19.81±0.89 | **0.020** | 0.315 | **0.008** | **0.014** |
| BMI, kg/m^2^ | 20.33±2.51 | 19.94±2.42 | 20.16±2.73 | 0.752 | 0.806 | 0.476 | 0.740 |
| Sex (Female) | 165 (62.74) | 26 (66.67) | 13 (41.94) | 0.061 | 0.635 | **0.039** | **0.025** |
| Grade (Freshman) | 206 (78.33) | 35 (89.74) | 21 (67.74) | 0.079 | 0.097 | **0.022** | 0.184 |
| Satisfied with the financial situation | 216 (82.13) | 34 (87.18) | 24 (77.42) | 0.563 | 0.436 | 0.282 | 0.522 |
| Pre-bedtime PESM-E | 140 (53.23) | 15 (38.46) | 24 (77.42) | **0.005** | 0.085 | **0.001** | **0.010** |
| Caffeine consumption | 144 (54.75) | 15 (38.46) | 16 (51.61) | 0.163 | 0.057 | 0.271 | 0.740 |
| Medication use | 71 (27.00) | 8 (20.51) | 5 (16.13) | 0.324 | 0.390 | 0.639 | 0.191 |
| **Sleep characteristics** | | | | | | | |
| PSQI, points | 4.51±2.13 | 3.56±2.28 | 4.87±2.28 | **0.006** | **0.003** | **0.006** | 0.430 |
| Bedtime | 23:53±0:34 | 23:29±0:26 | 24:08±0:28 | **<0.001** | **<0.001** | **<0.001** | **0.016** |
| Wake up time | 07:07±0:23 | 07:00±0:21 | 07:08±0:22 | 0.100 | **0.044** | 0.067 | 0.619 |
| TIB, hours | 7.23±0.66 | 7.52±0.62 | 6.99±0.59 | **0.001** | **0.001** | **<0.001** | 0.068 |
| Short sleep duration | 137 (52.09) | 13 (33.33) | 17 (54.84) | 0.079 | **0.029** | 0.071 | 0.772 |
| Sleep efficiency, % | 90.85±7.84 | 91.03±7.12 | 92.86±7.91 | 0.272 | 0.963 | 0.196 | 0.110 |
| SOL>30min | 23 (8.75) | 1(2.56) | 5 (16.13) | 0.151 | 0.310 | 0.081 | 0.317 |
| **Mood characteristics** | | | | | | | |
| BAI, points | 5.02±5.48 | 3.85±5.07 | 6.74±5.88 | **0.018** | 0.058 | **0.005** | 0.063 |
| BDI, points | 7.74±6.09 | 4.82±4.81 | 9.97±7.49 | **0.002** | **0.003** | **0.001** | 0.131 |

Abbreviations: BAI, Beck Anxiety Inventory; BDI, Beck Depression Inventory; BMI, body mass index; PESM-E, prolonged electronic screen media use for entertainment; PSQI, Pittsburgh Sleep Quality Index; SOL, sleep onset latency; TIB, time in bed. P_1_: Morningness vs. Intermediateness. P_2_: Morningness vs. Eveningness. P_3_: Intermediateness vs. Eveningness. P-values in bold (P<0.05) indicated a statistically significant difference.
